# The flavokawains: uprising medicinal chalcones

**DOI:** 10.1186/1475-2867-13-102

**Published:** 2013-10-22

**Authors:** Nadiah Abu, Wan Yong Ho, Swee Keong Yeap, M Nadeem Akhtar, Mohd Puad Abdullah, Abdul Rahman Omar, Noorjahan Banu Alitheen

**Affiliations:** 1 Faculty of Biotechnology and Biomolecular Science, Universiti Putra Malaysia, 43400 Serdang, Malaysia; 2 Bright Sparks Unit, University Malaya, 53500, Kuala Lumpur, Malaysia; 3 The University of Nottingham Malaysia Campus, 43500 Semenyih, Malaysia; 4 Institut of Bioscience, Universiti Putra Malaysia, 43400 Serdang, Malaysia; 5 Faculty of industrial Sciences & Technology, Universiti Malaysia Pahang, Lebuhraya Tun Razak 26300, Kuantan, Pahang, Malaysia

**Keywords:** Anti-cancer, Anti-inflammation, Apoptosis, Flavokawain, Kava-kava

## Abstract

Plant-based compounds have been in the spotlight in search of new and promising drugs. Flavokawain A, B and C are naturally occurring chalcones that have been isolated from several medicinal plants; namely the *piper methysticum* or commercially known as the kava-kava. Multiple researches have been done to evaluate the bioactivities of these compounds. It has been shown that all three flavokawains may hold promising anti-cancer effects. It has also been revealed that both flavokawain A and B are involved in the induction of cell cycle arrest in several cancer cell lines. Nevertheless, flavokawain B was shown to be more effective in treating *in vitro* cancer cell lines as compared to flavokawain A and C. Flavokawain B also exerts antinociceptive effects as well as anti-inflammation properties. This mini-review attempts to discuss the biological properties of all the flavokawains that have been reported.

## Introduction

For centuries, mankind have always opted natural products as an important companion in aiding illnesses and health-related diseases [1]. This is generally because it is relatively safe, cost-friendly and very diverse [2,3]. Kava-kava is a plant scientifically known as *piper methysticum* and it belongs to the pepper family *piperaceae*[4]. It is physically identified as a shrub and can be found in various parts of the world, mainly in the south pacific region [4]. The use of this plant in medicinal practices has been dated back to the eighteenth century [4]. It was found that kava plant acts as a diuretic and also as a mildly narcotic muscle relaxant [4,5]. Kava-kava naturally has a strong odor and a pungent taste, and is wildly cultivated among the Pacific Islanders [4]. A number of literatures have reported a variety of therapeutic properties in this plant species, such as anti-inflammatory, anti-cancer, anti-oxidant and hepatoprotective properties [4-6].

There are two major phytochemicals present within this plant; the kavalactones and chalcones [5,7]. Chalcones are a set of molecules derived from flavonoids and are also known as benzalacetophenone or benzylidene acetophenone [8,9]. The basic molecular structure of chalcones includes two aromatic rings linked by an unsaturated three carbon bridge [8]. The sources of chalcones are mainly from edible plants or can be readily synthesized by the Claisen-Schmidt condensation method [9-11]. This class of molecules has been found to possess several important bioactivities such as anti-inflammatory, anti-oxidant, anti-fungal, anti-angiogenic and anti-tumoral activities [11-15]. Chalcones in the kava plant can be recognized by their yellow appearance and are named flavokawains [5]. There are three types of Flavokawains that have been identified; Flavokawain A, Flavokawain B and Flavokawain C [16]. These compounds have similar backbones with different side chains [16]. Flavokawain A is the largest chalcone constituent with a 0.46% percentage in the kava extracts [16]. Flavokawain B follows this with 0.015% and Flavokawain C with 0.012% [16]. Among the three flavokawains, only flavokawain B has been extensively studied on. Table 1 summarizes the physical properties of Flavokawain A, B and C as well as depicting the molecular structure. Flavokawain B is scientifically known as 6’-hydroxy-2’,4’-dimethoxychalcone [17]. This compound was first reported to be found in the roots of *Piper methysticum*[18]. It was later found in other species as well such as *Aniba riparia*, *P*.*triangularis var*.*palloda* and *didymocarpus corchorijolia*[17]. Table 2 reports on the presence of the flavokawains in several plant species.

**Table 1:** **A summary of the properties of all three Flavokawains based on the findings of Dharmaratne *et al*.,**[16]

**Properties**	**Flavokawain A**	**Flavokawain B**	**Flavokawain C**
Molecular weight	314.33 g mol^−1^	284.31 g mol − 1	300.31 g mol^−1^
Molecular formula	C_18_H_18_O_5_	C_17_H_16_O_4_	C_17_H_16_O_5_
Molecular structure			
Physical appearance	Yellow crystalline	Yellow crystalline	Yellow crystalline
Melting point	111-115°C	96-98°C	185-188°C

**Table 2:** Plant species that have been reported to contain the Flavokawains

**Family**	**Flavokawain**	**References**
***Zingiberaceae***		
*Alpinia pricei Hayata*	Flavokawain B	[19]
*Alpinia pricei rhizome*		[20]
***Kava***		
*Piper methysticum*	Flavokawain A	[17]
*P*. *triangularis var*. *pallida*	Flavokawain B	[17]
*Didymocarpus corchorijolia*	Flavokawain C	[17]
*Aniba riparia*		[21]
***Annonaceae***		
*Goniothalamus gardneri*	Flavokawain A	[22]

## Flavokawain A

### Anti-inflammatory activity

The anti-inflammation activity of flavokawain A was examined by Kwon *et al*. in 2013 [23]. Kwon *et al*. conducted the research by inducing inflammation via LPS-stimulated RAW 264.7 cells [23]. Flavokawain A significantly suppressed the expression of iNOS and COX2. This is most likely accomplished by the inhibition of NF-ΚB pathway [23]. The halted production of these proteins also subdued the expression of NO and PGE_2_[23]. Upstream of iNOS and COX2, the activation of JNK and p38 MAPK were also inhibited. Additionally, flavokawain A inhibited the activation of AP-1 pathway upon the induction with LPS [23]. Moreover, flavokawain A also suppressed the expression of several pro-inflammatory cytokines including IL-6, IL-1B and TNF- **α**[23].

### Anti-cancer activity

Flavokawain A has been found to possess potential anti-cancer properties. For instance, in a research conducted by Tang *et al*. in 2008 [24], flavokawain A affected the cell cycle regulation of a p53-wild-type bladder cancer cell line (RT4). It was found that flavokawain A increased the amount of p21 and p27 cell cycle regulatory proteins and induced a G1 arrest [24]. Furthermore, it was discovered that in p53-mutant-type T24 cells, flavokawain A induced a G2/M arrest instead [24]. Tang *et al*. [24] suggested that the molecular mechanism underlying this occurrence was through the reduction of inhibitory kinases, Myt1 and Wee1 [24]. In a similar study conducted by Zi *et al*. in 2005, [7] flavokawain A was shown to execute a mitochondria-dependent apoptosis in T24 cells. Flavokawain A was proven to induce apoptosis in T24 bladder cancer cell line through the dependency on Bax, a mitochondrial protein [7]. The study reported that this compound also cleaved caspase 3, caspase 9 and poly- (ADP-ribose) polymerase to induce apoptosis in a dose-dependent manner [7]. This notion was also supported when there were changes in the mitochondrial membrane potential that lead to the release of cytochrome C [7]. Moreover, the ratio of Bax/Bcl-xl changed upon the treatment of flavokawain A. The ratio increased gradually until 650% in a time dependent manner [25]. Expectantly, this compound also decreased the level of X-linked inhibitor of apoptosis (XIAP) and survivin in T24 cells [25]. In an in-vivo xenograft model of bladder cancer, flavokawain A significantly decreased the tumor volume by approximately 57% [7].

## Flavokawain B

### Anti-inflammatory activity

The anti-inflammatory property of flavokawain B was assessed by a study conducted by Lin *et al*. in 2009 [25]. The study tested this compound against LPS-induced RAW 264.7 cells [25]. It was found that flavokawain B had an impressive effect in inhibiting nitric oxide production with an IC_50_ of 9.8 μM, lesser than the positive control used, curcumin [25]. Additionally, flavokawain B also inhibited PGE2 in a dose-dependent manner [25]. Similarly, these effects were also seen in the inhibition of TNF-**α**, a cytokine that is regularly involved in inflammation [25]. Furthermore, further analysis on the alteration of iNOS and COX-2 protein were also measured. Flavokawain B was found to inhibit the expression of both proteins depending on the concentration. To further clarify the activated inflammatory pathway upon the exposure of flavokawain B, NF- **κ**B – related proteins were tested [25]. Results showed that flavokawain B inhibited **κ** activation by degrading the inhibitory subunit IkB**α** and inhibiting the activation of I**κκ**[25]. Furthermore, flavokawain B has been found to significantly inhibit COX-I at 100 μg/ml, as well as COX-II enzyme [26].

### Antinociceptive activity

Two studies by Mohamad *et al*. [27,28] proved that flavokawain B has the potential to be developed into an antinocicepetive drug. Through the acetic acid-induced abdominal writhing test, it was discovered that at 128.6 mg/kg, the positive control, acetylsalicylic acid exhibited the same effect as flavokawain B when the dose was 10 mg/kg [27]. This indicates that flavokawain B was 68 fold more effective than acetylsalicylic acid [27]. This indictment also suggests that the activity of flavokawain B may involve inhibition of cyclooxygenases or lipooxygenases [27]. Moreover, flavokawain B was more effective when it is being administered intraperitoneally rather than orally by seven-fold [27]. To further elucidate the antinociceptive activity of flavokawain B, Mohamad *et al*. conducted another study and suggested that flavokawain B operates via the activation of the NO-cGMP-PKC-ATP-sensitive K + channels pathway [28].

### Anti-cancer properties

Researches in treating cancer, and better yet, preventing this disease has been significantly expanding. To date, the number of treatments varies and ranges from radiotherapy to herbal, alternative medicine. The most opted way of treating neoplasm is by administering drugs to the patient [29]. Flavokawain B has been found to be cytotoxic towards several important cancer cell lines. The most recent study was done by Ji *et al*. whom discovered the cytotoxic effects of flavokawain B on osteosarcoma cell lines [30]. This study revealed that flavokawain B exuded an apoptotic effect on these osteosarcoma cancer cell lines via the activation of caspase 3/7, caspase 8 and caspase 9 [30]. Furthermore, flavokawain B was also shown to down-regulate several anti-apoptotic proteins such as Bcl-2 and survivin [30]. Consequently, this compound also up-regulated various pro-apoptotic proteins including Bax, Fas and Puma [30]. Moreover, it was also observed, that flavokawain B induced a G2/M arrest by increasing Myt1 levels and concomitantly decreasing cdc2, cyclin B1 and cdc25c levels [30]. Interestingly, it has also been noted that flavokawain B decreased the migration and invasion ability of osteosarcoma cell lines [30]. This attribute is a favorable property especially in treating highly metastastic cancer cells.

Oral carcinoma is becoming more prevalent, especially in the South and Pacific Asia region [31]. Flavokawain B was shown to be cytotoxic towards the HSC-3, A-2058, Cal-27 and A-549 cell lines [19]. In normal gingival fibroblast cell line interestingly, flavokawain B showed very minimal cytotoxic effect [19]. Additionally, this compound was proven to cause a cell cycle arrest in the HSC-3 cell line. The number of cells in the G2/M phase increased in comparison with the control upon 12 hours of post-treatment [19]. The arrest in the G2/M phase may involve the inhibition of several important cyclin/cdk complexes. Hseu *et al*. in 2012, proposed that flavokawain B was involved in the inactivation of Cdc2, Cdc25c, Cyclin A and Cyclin B1 in HSC-3 cell line. Furthermore, it was also proven that this compound caused a shift in the mitochondrial membrane permeabilization potential, indicating that treated cells had a compromised mitochondria [19]. Furthermore, mitochondrial-related proteins were also regulated upon the induction of flavokawain B. Cytochrome c for instance, was found to be upregulated in the cytosol in a dose-dependent manner [19]. Moreover, flavokawain B also induced apoptosis in human oral adenoid cystic cancer, ACC-2 [32]. Flavokawain B was shown to induce G2/M arrest and induce the release of cytochrome c [32]. This subsequently lead to the activation of caspase 3 and the cleavage of the PARP enzyme [32].

Apart from HSC-3 cell line, flavokawain B can also be cytotoxic towards synovial sarcoma cell lines [33]. A study by Sakai *et al*. in 2011 [33], used two synovial sarcoma lines, SYO-1 and HS-SY-II to test for the cytotoxic activity of flavokawain B. Upon treatment, both the SYO-1 and HS-SY-II cell lines exhibited pro-apoptotic morphology such as cell membrane blebbing and cell shrinkage [33]. It is suggested that flavokawain B impeded cell growth in a concentration-dependent manner [33]. The increment of expression of caspase 8, 9 and 3/7 implied that flavokawain B activates the death-receptor and mitochondrial-mediated apoptotic mechanism [33]. Moreover in flavokawain B-induced SYO-I and HS-SY-II cells, the expressions of death receptor 5, Bim and Puma were up-regulated while the expression of survivin, an anti-apoptotic protein was down-regulated [33]. Another pro-apoptotic protein, Bak was also up-regulated when treated with flavokawain B at a concentration of 7.5 μg/ml [33].

Flavokawain B was also found to induce apoptosis in several prostate cancer cell lines while having insignificant effects on the normal prostate cell line [34]. This predicament is based on the morphology changes that occur in flavokawain B-treated cells which include, cell shrinkage, cell blebbing, nuclear fragmentation and condensation [34]. Additionally, Tang *et al*. [34] also discovered that flavokawain B induced apoptosis by the activation of several caspases in DU145 and PC-3 cell lines [34]. The cleavage of PARP, a pro-apoptotic protein was also seen in DU145 and PC-3 cell lines when induced with flavokawain B [34]. Besides, this compound increased the expression of several pro-apoptotic proteins, Bim, Bax and Puma, and conversely decreased the expression, of XIAP and survivin, both are anti-apoptotic proteins [34]. Interestingly, Li *et al.,* 2012, conducted another study regarding the effects of flavokawain B on several other prostate cancer-related cell lines such as LAPC4, C4-2B, LNCaP, 22RV1 and WPMY-1 [35]. This study showed that flavokawain B downregulated AR protein expression in all cell lines [35]. Additionally, flavokawain B also downregulated the expression of the AR target protein, PSA [35]. At the transcription level, flavokawain B significantly decreased the mRNA expression of AR, PSA and TMPSS2 in LNCaP and C4-2B cell lines [35]. It wasl aslo deiscovered that, flavokawain B decreased the level of AR through the downregulation of the SP1 protein expression [35].

Additionally, flavokawain B was also found to inhibit HCT116, a colon cancer cell line [6]. Kuo *et al*. [6], reported that flavokawain B induced an anti-proliferation state of HCT116 cells when treated with 5–50 μM of the compound. This observation was supported when the level of cleaved-PARP protein was noticeable at 50 μM of treatment [6]. The mitochondrial-induced apoptosis pathway was proposed because of the loss of the mitochondrial potential, release of cytochrome c and translocation of Bak [6]. Kuo *et al*. also found that GADD153, a marker for endoplasmic reticulum stress, was significantly upregulated. This further confirms the mitochondria-mediated apoptosis [6]. Additionally, BCL-2, an anti-apoptotic protein, downstream of GADD153 was downregulated when induced with flavokawain B [6]. Furthermore, it was discovered that flavokawain B increased the level of ROS and p38, both contributing to the execution of apoptosis [6]. Likewise to Hseu *et al*. 2012, flavokawain B was also found to induce a G2/M arrest in HCT116 cells [6].

Another study was conducted by An *et al*. in 2012, addressed the anti-neoplastic activity of flavokawain B on non-small cell lung cancer, H460 cells. The findings were similar to the other studies conducted; flavokawain B induced a mitochondrial-dependent apoptosis pathway [36]. This study showed that flavokawain B induced the release of cytochrome c and deregulated the BcL-xL/Bax ratio [36]. Likewise to other studies, it was also proven that flavokawain B down-regulated the anti-apoptotic proteins, XIAP and survivin [36]. Additionally, it was found that flavokawain B activated the MAPK pathway and subsequently the JNK-mediated apoptotic pathway [36]. The effects of flavokawain B on uterine leiomyosarcoma cell lines were tested by Eskander *et al.* 2012. Similar to other studies, this study also concluded that flavokawain B induced a G2/M arrest in treated cells [37]. Furthermore, also in accordance with other findings, flavokawain B up-regulated several pro-apoptotic proteins such as death receptor 5, Puma and Bim, and also down-regulated IAP and survivin [37].

Another recent study on the anti-cancer mechanism of flavokawain B was conducted on squamous carcinoma cells, KB cells [38]. Lin *et al.* in 2012, designed the study to determine the apoptotic, anti-proliferative activity and anti-metastatic activity of flavokawain B on KB cells [38]. This study also prove that flavokawain B induced a G2/M arrest as evidenced by the reduction of cyclin A, cyclin B1, cdc2, cdc25c and increment of p53, p21 and wee1 [38]. The induction of apoptosis on the other hand, was substantiated by the activation of caspase 3,-8 and −9 as well as the cleavage of PARP [38]. Additionally, the levels of Bid and Bax were increased coupled with the decrement of Bcl2 [38]. As an anti-metastatic drug, flavokawain B remains promising as it inhibited the expression of matrix-metalloproteinase 9 and urokinase plasminogen activator [38].

Besides *in vitro*, analyses on *in vivo* model were also conducted by some studies [7,24,34]. A research by Tang *et al*. tested the compound flavokawain B on nude mice induced with DU145 cells. Expectantly, flavokawain B significantly reduces the growth of these tumors by approximately 67% [34]. The level of Bim was found to be increased in tumor lysates as compared to the untreated control [34]. Additionally, it was reported that there were no necropsy or growth irregularities in the treated mice [34]. The anti cancer in vivo effects of flavokawain B in KB xenograft models was also tested [38]. Flavokawain B significantly reduced the growth of the tumor as proven by the augmentation of apoptotic DNA fragmentation [38]. Li *et al.* 2012, also studied the interaction of flavokawain B on two patient-derived prostate cancer xenografts in mice. The study found that flavokawain B inhibited the growth of the tumor, reduced the expression of AR as well as the levels of serum PSA [35].

### Other properties

Additionally, a research conducted by Feroz *et al.* studied the interaction between flavokawin B and human serum albumin [39]. The efficacy of the pharmacokinetics and pharmacodynamics of a certain drug once it is inside the body is greatly dependent on its interaction with plasma protein [39]. It is well known that human serum albumin is a major player in the transport of various ligands [39]. As expected, flavokawain B was found to interact with human serum albumin similarly to other flavonoids [39]. The binding essentially comprises of hydrophobic interactions including hydrogen bonding [39].

## Flavokawain C

### Anti-cancer properties

Flavokawain C has been tested for its anti-cancer activities against several bladder cancer cell lines [7]. The results showed that flavokawain C induced an anti-proliferative and an apoptotic state in these cell lines (T24, RT4 and EJ cells) [7]. The IC_50_ of flavokawain C was promising as it was relatively low (≤17 μM) [7]. Additionally, in two liver cancer cell lines, L02 and HepG2, flavokawain C had a substantially higher IC_50_ value (<60 μM) as compared to the bladder cancer cell lines [40]. Nevertheless, further analysis should be done to determine flavokawain C’s effects on normal cell lines.

## Comparison of the induction of apoptosis

Through the findings of these studies, it can be suggested that the anti-cancer action of flavokawain A is dependent on the p53 status of the cancer cells. In the p53-wild type cancer cells, flavokawain A is more likely to induce a G1 arrest instead of a G2/M arrest in the p53-mutant cells. This statement needs further proof to definitively conclude whether the effect of flavokawain A is p53-dependent. Majority of the studies conducted suggest that flavokawain B induces apoptosis both through the mitochondria-dependent pathway and death-receptor pathway. Additionally, flavokawain B induces G2/M arrest in most of the tested cell lines regardless of the p53 status, unlike flavokawain A.

Since inflammation plays a major role in cancer progression, the anti-inflammatory effects of flavokawain A and B should also be considered in elucidating its anti-cancer activity. Flavokawain A and B inhibited the NF-κB pathway and subsequently the JNK pathway. This suggest that flavokawain A and B may also induce TNF-α-mediated apoptosis [41]. The JNK pathway may also induce changes in the mitochondria membrane potential [41,42]. This may consequently lead to the release of cytochrome c and the activation of caspases as observed in several studies [42]. Additionally, most of the studies revealed that there were changes in the expression level of some mitochondrial-related proteins, especially in the BCL2 family [30,33]. A summary of the IC_50_ values of all three flavokawains in various cell lines is provided in Table 3.

**Table 3:** **A summary of *in vitro *** **IC**_**50 **_**of Flavokawain A, B, C in selected cancer cell lines**

**Flavokawain**	**Cancer cell lines**	**IC**_**50 **_**μM**
**Flavokawain A**	T24	~17
	RT4	~17
	EJ	~17
	L-02	n.c.
	HepG2	n.c.
**Flavokawain B**	KB	20.05 ± 0.4
	MCF-7	49.30 ± 1.8
	Ca Ski	31.10 ± 1.3
	HCT116	~25
	A549	~25
	HFW	~25
	NIH3T3	~25
	MRC5	17.2 ± 0.5
	T24	4.39-8.80
	RT4	~18
	EJ	~9-18
	LAPC4	32
	LNCaP	48.3
	PC-3	6.20
	DU145	3.90
	WPMY-1	**2.25**
	22RV1	**16.6**
	C4-2B	**2.2**
	L-02	35.15 ± 2.56
	HepG2	62.38 ± 5.04
	HSC-3	~18
	Cal-27	~27
	A-2058	~18
	SYO	~9-18
	HS-SY-II	~18-27
	H460	18.2
	ACC-2	4.69 ±0.43
	T24	~8-17
**Flavokawain C**	RT4	~1.5-17
	EJ	~8.33
	L-02	57.04 ± 2.32
	HepG2	59.48 ± 2.72

[6,7,19,24,25,33-35,38,40].

## Structure-activity relationship

All three flavokawains share a similar backbone bearing a 2’-hydroxy-4’, 6’-dimethoxychalcone [16]. The differences lay in the R-4 position; flavokawain A contains a methoxy group, flavokawain B only retains the designated hydrogen, and flavokawain C with an extra hydroxyl group [16]. All three molecules have the same physical structure except for the melting point. In terms of bioactivity, based on evidenced studies, both flavokawain A and flavokawain B possess anti-inflammatory activity at almost similar concentrations [23,25]. The difference in the methoxy group did not account for a substantial difference. The difference in the IC_50_ of the flavokawains against three bladder cancer cell lines (T24, RT4, and EJ) and two liver cancer cell lines (HepG2 and LO2) can be assessed to determine the structure-activity relationship. The IC_50_ of flavokawain A is slightly higher than the other two flavokawains in the bladder cancer cell lines [7,40]. In the liver cancer cell lines however, flavokawain A did not have an IC_50_ value, unlike flavokawain B and C [40]. This is probably due to difference in the side chain. Bearing a methoxy group could introduce steric hindrance and possibly explain the decrease in anti-cancer activity as compared to the other two flavokawains [43].

## Toxicity

The kava plant has long been used in traditional practices as an herbal tonic [4]. Nevertheless, there have been reports claiming that the flavokawains may cause hepatotoxicity [44]. This is due to the cases that kava extracts may cause several hepatic failures in consumers [45]. A study by Zhou *et al*. [46] also suggested that flavokawain B is a hepatotoxin in the kava extracts [46]. However, there have been no reports on any liver damages when *in vivo* studies were done [34]. Teschke *et al*. [44] has asserted that the liver toxicity cases were only reported in the South pacific region and this is probably due to the tropical humidity and temperature. The kava plants may have been contaminated by mould hepatotoxins instead [47]. Teschke *et al*. [44] also stated that the level of flavokawain B in the kava extracts is too low to be causing liver injury [47]. There is a need to determine the acute and subchronic toxicity *in vivo* to further clarify whether the flavokawains are safe to be consumed and developed into the pharmaceutical line.

## Conclusion

The Flavokawains are becoming more favorable in becoming anti-cancer agents candidates. Most of the studies reveal that the flavokawains induce apoptosis instead of necrosis in cancer cell lines. This is generally a promising factor if it is further developed into the commercial line. Flavokawain A and flavokawain B has been found to interact with several important molecular proteins and signaling pathways. Additonally, the flavokawains (A and B) were shown to inhibit the NF- **κ**B pathway and the inflammatory process *in vitro*. The flavokawains also exhibited anti-cancer activities in xenograft models. Even though there have been reports regarding the liver toxicity induced by flavokawain B, this assertion has been refuted. The flavokawains still remain a potential group of molecules that can be used as anti-cancer agents. However, further in depth studies must be done in order to fully understand the mechanism underlying flavokawain’s potent activity.

## Competing interests

The authors declare that they have no competing interests.

## Authors’ contributions

NA did the literature search and drafted the manuscript. NBMA, WYH, SKY, MNA, MPA, ARO conceived the idea, contributed to the discussion and edited the manuscript. All authors read and approved the final manuscript.
